# DJ-1 is activated in medulloblastoma and is associated with cell proliferation and differentiation

**DOI:** 10.1186/1477-7819-12-373

**Published:** 2014-12-05

**Authors:** Jia-ping Lin, Bin-cai Pan, Bin Li, Yang Li, Xiao-ying Tian, Zhi Li

**Affiliations:** Department of Neurosurgery, the First Affiliated Hospital, Sun Yat-sen University, 58, Zhongshan Road II, Guangzhou, 510080 China; Department of Pathology, Tongjiang Hospital of Guangdong, Nanguo Road East, Shunde District, Foshan, 528300 China; Department of Pathology, The First Affiliated Hospital, Sun Yat-sen University, 58, Zhongshan Road II, Guangzhou, 510080 China; School of Chinese Medicine, Hong Kong Baptist University, 7, Baptist University Road, Kowloon Tong, Hong Kong, China

**Keywords:** Medulloblastoma, DJ-1, PI3K/Akt pathway, PTEN, Prognosis

## Abstract

**Background:**

DJ-1 is a key regulator in human tumorigenesis, including brain malignancies. The mechanisms by which DJ-1 contributes to the pathogenesis of medulloblastoma (MB) remain unclear, and its impact on the prognosis for patients with MB has not been identified. The aim of this study was to determine whether the DJ-1 protein is associated with tumorigenesis of MBs, and whether DJ-1 is a valuable factor for predicting the prognosis of patients with MB.

**Methods:**

We collected 66 pairs of MB and adjacent normal cerebellum samples. Expression of DJ-1, Ser ^473^-phosphorylated-Akt (p-Akt), PTEN, and Ki-67 (MIB-1) was detected by immunohistochemical staining, and the correlation of these immunostaining results with the clinicopathological features of patients with MB was determined.

**Results:**

High DJ-1 expression (48.5%, 32/66) in tumor cells of MBs was significantly associated with the classic MB variant (*P* = 0.003), high proliferative activity (*P* = 0.002) and undifferentiated tumor (*P* = 0.001), whereas high p-Akt expression (56.1%, 37/66) was associated with tumor metastasis stage (*P* = 0.007), undifferentiated tumor (*P* = 0.007), and high-risk tumor (*P* = 0.002). High DJ-1 expression also correlated with high p-Akt expression and high MIB-1 index. However, only high levels of DJ-1(*P* = 0.009) and high MIB-1 index (*P* = 0.001) were strong independent prognostic factors associated with worse overall survival.

**Conclusions:**

Although the validity of the preliminary data in this study needs to be confirmed by a larger number of cases, our study indicates that DJ-1, PTEN, and p-Akt might play important roles in cell proliferation and differentiation of MBs. The evaluation of expression of DJ-1 and related proteins might be useful for predicting the prognosis of patients with MB.

## Background

Medulloblastoma (MB) is the most common malignant tumor of central nervous system in children [1], while it rarely occurs in adults [2]. Although multimodality treatment regimens, including surgery, radiotherapy (RT), and chemotherapy, have greatly improved disease outcome, about one-third of patients with MB remain incurable. Moreover, the 5-year disease survival rate is only 36% for patients with MB with tumor dissemination and recurrence [3, 4]. The accumulated studies in MB have come to a consensus that MB can be classified into four core subgroups: WNT (Wingless), SHH (Sonic hedgehog), Group 3, and Group 4 [5]. Each of these subtypes has a distinct molecular profile and genomic defects, which are associated with varied clinical parameters and patient outcomes. However, despite extensive investigation, the mechanism underlying MB progression has not been fully elucidated. More precise prognostic predictors and more effective therapies for MBs are therefore required.

Recently, research evidence has suggested that DJ-1 plays a role in human tumorigenesis. DJ-1 is a 189 amino acid protein, and was originally identified as an oncogene that, in combination with H-RAS, can transform mouse NIH3T3 cells [6]. The DJ-1 protein has been found in various malignant tumor cells, including prostate cancer, non-small cell lung cancer, laryngeal cancer, ovarian carcinoma, and cervical cancer, where it plays a role in increasing cell proliferation and metastasis [7–10]. However, the effect of DJ-1 on the development and progression of MB has not yet been investigated.

DJ-1 is considered to contribute to oncogenesis by upregulating protein kinase B (PKB/Akt)-mediated cell survival [6, 11]. Akt is a 57-kDa serine/threonine kinase, and is a central mediator involved in the signal transduction of various growth-controlling pathways that involve phosphatidylinositol 3′-kinase (PI3K). PI3K, activated by growth factors, catalyzes the phosphorylation of phosphatidylinositol (4,5)-biphosphate (PIP2) to phosphatidylinositol (3,4,5)-triphosphate (PIP3). PIP3 in turn recruits 3-phosphoinositide-dependent kinase (PDK), which phosphorylates and activates Akt [12]. Phosphorylated Akt (p-Akt) plays a key role in multiple signaling pathways, including cell proliferation, apoptosis, and transcription [13]. Basic research has demonstrated that DJ-1 could antagonize the tumor suppressor PTEN to inhibit the activity of the *PTEN* gene and finally promote the proliferation of tumor cells [11]. PTEN is a key negative regulator of the PI3K-protein kinase B signaling pathway. In MBs, loss of heterozygosity of chromosome 10q is frequent at the position where PTEN is located (10q23.31) [14, 15]. Dysregulation of PTEN was found to contribute to overactivation of the PI3K/Akt signaling pathway [16, 17]. Thus, we hypothesized that DJ-1 might contribute to the progression of MB by regulating the PTEN and Akt pathways. However, we could not find any report about the relationship between DJ-1 and tumorigenesis of MBs, or the mechanisms involved.

In this study, we first examined the DJ-1, PTEN, and p-Akt expression in surgical MB tissue specimens and paired tumor-adjacent tissue specimens. The aim of this study was to determine whether the DJ-1 protein is associated with tumorigenesis of MBs, and if it would be a valuable factor for predicting the prognosis of patients with MB.

## Methods

### Patients and clinical management

In retrospective study, we examined 66 pairs of paraffin wax-embedded MB and adjacent normal cerebellum samples collected from the Pathology Department of the First Affiliated Hospital of Sun Yat-sen University and Tongjiang Hospital of Guangdong during the period of 2003 to 2012. None of the patients had received chemotherapy or RT before surgical treatment. All experimental protocols were carried out with the approval of the Committee on Use of Human & Animal Subjects in Teaching and Research of Sun Yat-sen University, according to the Helsinki Declaration.

All patients had undergone midline suboccipital craniectomy to resect the tumor completely or partially. Extent of resection was defined on the basis of surgical reports and/or postoperative images. Patients received different treatment protocols after surgery, based on age stratification. All patients under 3 years of age received chemotherapy after surgery with standard regimens consisting of six course of temozolomide, vincristine, and cisplatin. Patients aged over 3 years initially received RT. Postoperative RT was conducted using craniospinal irradiation (CSI), with a subsequent boost to the posterior fossa. Patients received a total dose ranging from 40 to 54 Gy. After RT, six courses of standard chemotherapy with temozolomide, vincristine, and cisplatin were also used for these patients. Imaging inspection was repeated at intervals of 3 to 6 months to evaluate treatment response, metastasis, and tumor recurrence. All 66 patients were given a follow-up investigation, and follow-up was terminated in March 2013.

### Histopathological subtype

All the samples were re-evaluated according to the criteria of the WHO classification [18] by two experienced pathologists, with differences resolved by careful discussion. Of the 66 case of MB, 50 (75.7%) were classified as classic MB, 14 (21.2%) as the desmoplastic variant, 1 (1.5%) as the large cell/anaplastic variant, and 1 as MB with myogenic differentiation (previously termed medullomyoblastoma).

### Immunohistochemical staining and scoring

Serial sections 4 μm thick were cut from eah tissue block and mounted on aminopropyltriethoxysilane (APES)-coated glass slides. After routine preparation of slides, the sections were incubated with one of the following antibodies: rabbit anti-human monoclonal PTEN antibody (1:100 dilution; D4.3), p-Akt (Ser 473) rabbit monoclonal antibody (1:25; D9E) (both Cell Signaling technology, MA, USA), rabbit anti-human polyclonal DJ-1 antibody (1:100; FL-189; Santa Cruz Biotechnology, SantaCruz, CA, USA), or Ki-67 (1:100 dilution; MIB-1; Dako Co., Glostrup, Denmark) for 60 min, respectively. Slides were processed using a ChemMate Envision/horseradish peroxidase kit (Dako) for 30 min at room temperature, followed by development with diaminobenzidine (DAB) for visualization. Positive controls consisted of biopsies of prostate tissue containing carcinoma and normal parenchyma (for DJ-1 and PTEN) and breast cancer (p-Akt). Negative controls were prepared by substituting non-immune serum for primary antibodies.

Immunohistochemical staining evaluation was conducted as described previously [19]. Scoring of the percentage of immunoreactive tumor cells was as follows: 0 (0%), 1 (1 to 10%), 2 (11 to 50%), and 3 (>50%). The staining intensity was visually scored and stratified as 0 (negative), 1 (weak), 2 (moderate), and 3 (strong). A final immunoreactivity score was obtained for each case by multiplying the percentage and the intensity score. The median score was 4.5. Thus, DJ-1, PTEN, and p-Akt protein expression levels were further classified as lower (when the score was less than 4.5) and higher (when it was greater than 4.5). In order to reduce variation, all immunohistochemical staining was separately evaluated and scored semiquantitatively by two pathologists without knowledge of the clinical data of patients. The final score for protein expression in each case was the mean of the scores given by the two observers.

The proliferative activity of tumor cells was evaluated by counting the Ki-67 (MIB-1) labeling index with image analysis (Olympus Cell D1; Soft Imaging System GmbH, Munster, Germany) counting at least 1000 tumor cells at high magnification (×40 objective) in fields with the largest number of positive cells. Tissues were considered to have a high MIB-1 index (MI) when the Ki-67 labeling value was greater than the mean Ki-67 labeling value of all 66 MBs.

### Statistical analysis

Statistical analysis was performed using SPSS software (version 14.0 for Windows; SPSS Inc., Chicago, IL, USA). Qualitative data are presented as number and percent. The χ^2^ was used for comparison between groups, and *P* < 0.05 was considered significant. Overall survival (OS) was counted (months) from the date of diagnosis to the date of death or last follow-up before study closure. Kaplan-Meier method and Cox regression were used for survival analysis.

### Ethics committee approval and patient consent

This study was approved by Sun Yat-sen University review board or ethics committee.

## Results and discussion

### DJ-1, p-Akt, and PTEN expression in MBs

**Figure 1:**
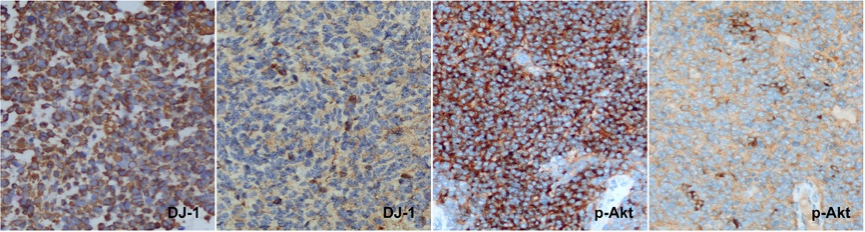
**Expression levels of DJ-1 and p-Akt in tumor cells with varying levels of positive percentage and intensity.** High and low expression of DJ-1 are shown on the left side, and high and low expression of p-Akt on the right side. (Original magnification × 400).

**Figure 2:**
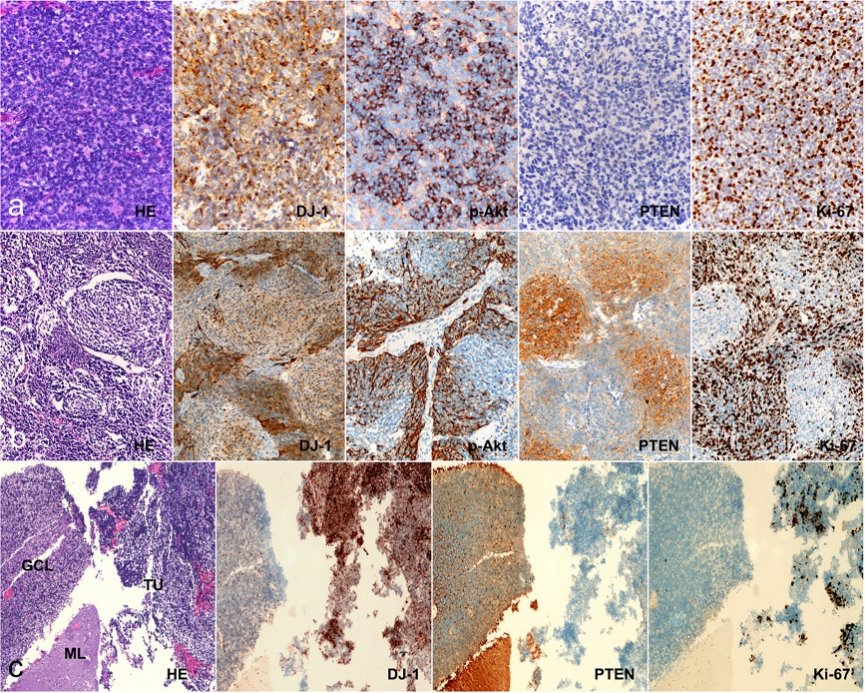
**Immunohistochemical analysis of DJ-1, PTEN, p-Akt, and Ki-67 expressionin MBs. (a)** In classic MBs, DJ-1 and p-Akt appeared to have diffused positive signal in tumor cells, but PTEN was negative in most cases. Prolifera**t**ive activity (Ki-67 labeling) was particularly prominent in areas with high expression of DJ-1 and p-Akt. **(b)** In desmoplastic MBs, high expression of DJ-1, p-Akt, and Ki-67 was observed in internodular areas with undifferentiated or poorly differentiated tumor cells; however, detectable signals of PTEN were observed in intranodular areas with neuronal differentiation. **(c)** Immunohistochemical analysis showed that expression of DJ-1 was significantly higher in tumor cells than in normal cerebellum, but PTEN was found to be expressed at significantly lower levels in tumor cells compared with adjacent normal cerebellum. ML, molecular layer of cerebellum; GCL, granule cell layer of cerebellum; TU, tumor. (Original magnification × 200).

All 66 MB samples showed detectable levels of DJ-1 and p-Akt in tumor cells with variations in positive percentage and intensity (Figure 1). However, DJ-1 and p-Akt expression was not present in adjacent normal cerebellar tissue. The areas with high DJ-1 expression were observed to almost overlap with those with p-Akt high expression. Proliferative activity was particularly prominent in areas with high expression of DJ-1 or p-Akt, which were 8.5% (32/66) and 56.1% (37/66), respectively. In the desmoplastic variant MBs, DJ-1 and p-Akt showed a staining pattern, in which the internodular (undifferentiated or poorly differentiated) areas had higher expression than the intranodular (neuronal differentiated) regions (Figure 2a, b). PTEN expression was observed in normal cerebellum, including the molecular layer and inner granule cell layer. However, PTEN protein level was significantly decreased in tumor cells, and low expression of PTEN was seen in all MB samples. We found that 84.8% (56/66) of samples, including all the classic MBs and four of the desmoplastic variant samples, showed no detectable or only weak levels of PTEN in tumor cells. A focal immunopositive PTEN signal was observed in the intranodular regions of the other 10 desmoplastic variant MBs (Figure 2c). In addition, the areas with high expression of p-Akt coincided with the regions with loss of PTEN expression.

### Correlation of DJ-1, p-Akt, and PTEN expression with the clinicopathological characteristics of medulloblastoma

We found statistically significant correlations of high DJ-1 protein expression with slightly older children and adults. Although MB is the most common in children, it rarely occurs in adults. In this study, several adult patients were collected and stratified into the study group of patient more than 3 years old. (*P* = 0.030), classic variant MBs (*P* = 0.003), high proliferative activity (*P* = 0.002), undifferentiated tumor (*P* = 0.001), and high-risk tumor (*P* = 0.031). High p-Akt expression was also associated with tumor metastatic stage (*P* = 0.007), undifferentiated tumor (*P* = 0.007), and high-risk tumor (*P* = 0.002). High p-Akt expression also correlated with high DJ-1 expression in tumor cells (*P* = 0.010). However, the expression levels of these proteins were not statistically associated with sex, tumor location, or size of residual tumor (Table 1).

**Table 1:** **Correlation between proteins expression and clinicopathological characteristics of patients with MB**

Clinicopathological characteristics	DJ-1 expression ^a^		p-Akt expression ^a^		Proliferative activity (Ki-67 value) ^b^
	Low (n = 34)	High (n = 32)	Low (n = 29)	High (n = 37)	
Age, year					
≤3 (n = 19)	13	6	7	12	25.28 ± 7.59
>3 (n = 47)	21	26	22	25	26.77 ± 8.74
*P* value	0.030		0.275		0.519
Sex					
Male (n = 43)	23	20	18	25	25.80 ± 7.31
Female (n = 23)	11	12	11	12	27.36 ± 10.23
*P* value	0.619		0.527		0.475
WHO histological subtype ^c^					
Classic (n = 50)	23	27	22	28	25.17 ± 7.75
Desmoplastic (n = 14)	11	3	7	7	30.03 ± 9.89
*P* value	0.003		0.536		0.055
Tumor location					
Fourth ventricle (n = 29)	16	13	10	19	26.65 ± 8.51
Outside fourth ventricle (n = 37)	18	19	19	18	26.11 ± 8.42
*P* value	0.556		0.065		0.795
Residual tumor size					
≤1.5 cm^2^ (n = 54)	29	25	24	30	25.75 ± 8.14
>1.5 cm^2^ (n = 12)	5	7	5	7	29.04 ± 9.38
*P* value	0.221		0.829		0.222
Metastatic status					
M0 (n = 12)	5	7	3	9	28.20 ± 7.39
M1 (n = 54)	29	25	26	28	25.93 ± 8.61
*P* value	0.221		0.007		0.401
Tumor risk					
Standard (n = 6)	2	4	1	5	30.50 ± 9.58
High (n = 60)	32	28	28	32	25.93 ± 8.25
*P* value	0.031		0.010		0.201
Differentiation level					
Undifferentiated (n = 39)	14	25	13	26	27.28 ± 8.82
Differentiated (n = 27)	20	7	16	11	25.00 ± 7.71
*P* value	0.001		0.007		0.281
DJ-1 expression					
Low (n = 34)			20	14	23.33 ± 6.22
High (n = 32)			9	23	29.54 ± 9.28
*P* value			0.001		0.002
p-Akt expression					
Low (n = 29)	20	9			25.13 ± 6.92
High (n = 37)	14	23			27.29 ± 9.38
*P* value	0.001				0.303

^a^χ^2^ test.

^b^One-way ANOVA.

^c^We did not carry out statistical analysis for the large cell/anaplastic variant and medullomyoblastoma because there were insufficient numbers of cases.

### Prognostic relevance of DJ-1 and p-Akt expression in MBs

**Figure 3:**
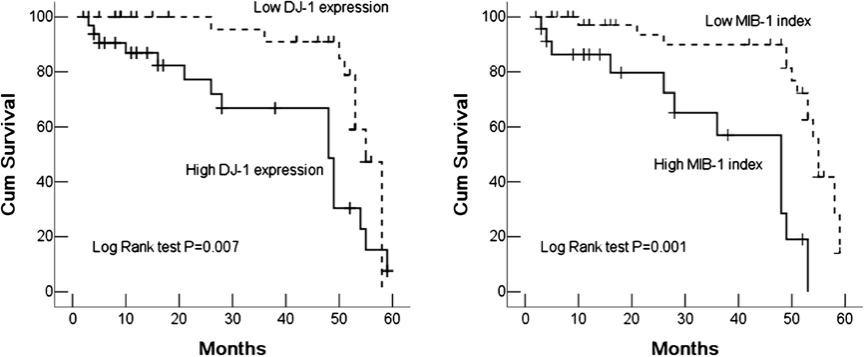
**Kaplan-Meier survival curves for DJ-1 expression and proliferative activity in tumor cells of MBs.** Patients with MB with high DJ-1 expression (left) or high MIB-1 index (right) had poorer OS.

The follow-up time for the 66 patients with MB was 2–59 months. The most recent follow-up showed that 40 patients were still alive, and the 5-year OS was approximately 60.6%. Kaplan-Meier survival analysis revealed that patients with MB with high DJ-1 expression had poorer OS than those with low DJ-1 expression (*P* = 0.007). Patients with MB tumors with high MI also had poorer prognosis than those with low MI (*P* = 0.001) (Figure 3). Univariate analyses of each factor with Cox log-rank analysis (Table 2) showed that the other clinicopathological parameters, including metastatic stage, histopathological subtype, and cell differentiation were not significantly associated with tumor prognosis. In multivariate analysis (Table 3), only high proliferative activity (*P* = 0.001) and high DJ-1 expression (*P* = 0.009) in tumor were independent predictors of OS for patients with MB.

**Table 2:** **Kaplan-Meier analysis for OS rate of patients with MB**

Clinicopathological characteristics	Mean survival time, months	95% CI, months	***P***value ^a^
Age, year			
≤ 3	44.96 ± 5.18	34.81 to 55.12	0.495
>3	47.72 ± 2.72	42.95 to 52.48	
Sex			
Male	47.16 ± 2.70	41.86 to 52.46	0.880
Female	46.60 ± 4.10	38.56 to 54.64	
WHO histological subtype^b^			
Classic	48.39 ± 2.41	43.66 to 53.12	0.263
Desmoplastic	41.39 ± 5.93	29.76 to 53.02	
Tumor location			
Fourth ventricle	46.35 ± 3.84	38.82 to 53.88	0.889
Outside fourth ventricle	47.66 ± 2.61	42.44 to 52.87	
Residual tumor size			
≤1.5 cm^2^	37.78 ± 6.14	25.73 to 45.83	0.052
>1.5 cm^2^	49.68 ± 2.28	45.19 to 54.16	
Metastatic status			
M0	42.08 ± 7.92	26.54 to 57.62	0.971
M1	47.45 ± 2.33	42.87 to 52.02	
Tumor risk			
Standard	46.64 ± 2.34	41.04 to51.24	0.062
High	23.75 ± 5.20	13.54 to33.95	
Differentiation level			
Undifferentiated	46.84 ± 3.03	40.89 to 52.79	0.823
Differentiated	46.87 ± 3.52	39.96 to 53.78	
DJ-1 expression			
Low	53.29 ± 1.79	49.77 to 56.81	0.007
High	40.28 ± 3.79	32.84 to 47.71	
p-Akt expression			
Low	47.76 ± 3.19	41.50 to 54.03	0.663
High	46.30 ± 3.23	39.97 to 52.63	

aLog rank test.

^b^We did not carry out statistical analysis for the large cell/anaplastic variant and medullomyoblastoma because there were insufficient numbers of cases.

**Table 3:** **Cox regression model for multivariate analyses of prognostic factor in MBs**

Variable	Hazard ratio	95% confidence interval	***P***value
Age (≤3 years *versus* >3 years)	0.396	0.140 to 1.118	0.080
Gender (male *versus* female)	1.421	0.485 to 4.160	0.521
WHO histological subtype (classic *versus* desmoplastic)	0.931	0.308 to 2.808	0.899
Tumor location (fourth ventricle *versus* outside fourth ventricle)	1.201	0.404 to 3.573	0.741
Residual tumor size (≤1.5 cm^2^ *versus* >1.5 cm^2^)	0.391	0.139 to 1.102	0.075
Metastatic status (M_0_ *versus* M_1_)	1.720	0.310 to 9.546	0.534
Tumor risk (standard *versus* high)	0.695	0.041 to 11.92	0.802
Differentiation (differentiated *versus* undifferentiated)	0.348	0.121 to 0.998	0.050
MIB-1 index (low *versus* high)	5.949	1.986 to17.814	0.001
DJ-1 expression (low *versus* high)	4.531	1.443 to14.223	0.009
p-Akt expression (low *versus* high)	0.765	0.217 to 2.698	0.678

MBs are a heterogenous group of highly malignant embryonal brain tumors [20]. For many years, MBs have been stratified into high-risk and standard-risk groups according to age, metastatic stage at diagnosis, and extent of surgical resection. Standard-risk patients are those diagnosed at ages over 3 years, have no metastases at presentation, and have manageable residual tumor (<1.5 cm^2^) after surgery. High-risk patients are those do not fulfill these criteria [21], and these patients should receive more intensive treatment. A recent study has shown that the combination of chemotherapy and RT has improved the 5-year survival rate, which is now 55% to 76% for high-risk patients and 70% to 80% for standard-risk patients [22]. However, in the present study, we found that there was no significant difference in OS of patients with different tumor risk, and tumor risk stratification was not an independent factor for predicting the prognosis of patients with MB. The different treatment protocols based on age stratification did not show any marked benefit for prognosis. These results indicate that risk-adapted treatments are not entirely suitable for all patients with MB. Hence, it is important to identify novel clinical and biological factors that can be used to predict treatment response and accurately select patients who may benefit from a more or less aggressive treatment, and to improve risk stratification.

The importance of DJ-1 in the progression of MBs has not been identified, although recent studies have demonstrated that the DJ-1 molecule is relevant to the various human tumors [20, 23–25]. In the human brain, DJ-1 has been observed in neurons [26] and non-neural cells [27]. DJ-1 has also been associated with autosomal recessive early-onset parkinsonism, with loss of function of DJ-1 leading to neurodegeneration [28]. In gliomas, the relationship between high DJ-1 expression and high-grade glioma has indicated that DJ-1 might play a role in the acceleration of glioma progression [20]. In the current study, we found the first evidence that high DJ-1 expression was closely correlated with undifferentiated tumor and active proliferation of tumors. Survival analysis revealed that high DJ-1 expression was significantly associated with low OS in patients with MB. Moreover, on multivariate analysis, high DJ-1 expression emerged as an independent factor influencing the prognosis of patients with MB. These results indicate that DJ-1 may promote tumor progression and could be an independent prognostic factor by influencing cell proliferation and differentiation. To date, there is still no conclusive evidence that measures of cell proliferation have clinical utility, and the relevant studies have provided conflicting data. Some studies have shown an association between a simple evaluation of mitotic count and survival for children with MB [29, 30]; however, survival analyses do not support the use of the Ki-67/MIB-1 index as prognostic indicator [31], although high bromodeoxyuridine (BrdU) index (>20%) appeared to indicated worse prognosis in one study [32]. In the present study, high MIB-1 index had a strong correlation with poor prognosis of patients with MB and may be an independent indicator of tumor. More importantly, high proliferative activity of tumor cells was associated with high expression of DJ-1 protein. These data suggest that the cell proliferation influencing the prognosis of patients with MB might be modulated, at least partly, by the pathway involving the DJ-1 molecule.

DJ-1 is considered to contribute to oncogenesis by upregulating PKB/Akt-mediated cell survival [6, 11]. However, in this study, however, we did not find an association between high p-Akt expression by tumor and high MIB-1 index of tumor cells, although high p-Akt expression was observed to correlate closely with tumor differentiation and metastasis.

Consistent with this result, p-Akt expression was not a prognostic indicator for predicting the prognosis of patients with MB. On the one hand, activated Akt was expressed at much higher levels in MB tumors compared with normal human cerebellum, indicating that p-Akt may be up-regulated in tumourigenesis and tumor progression of MBs, such as modulating tumor differentiation and promoting metastasis. On the other hand, activated Akt is an essential, but not the only, pathway in promoting cell proliferation of MBs.

In addition, in the current study, we found that the areas having high DJ-1 expression always overlapped with those having p-Akt high expression.

These results suggest that DJ-1 does indeed have a role in modulating the Akt pathway in MBs, but it might not be the only factor to influence activated Akt in tumor progression. Recent studies have demonstrated that several growth factor receptors involving the PI3K/Akt cascade in their signal transduction are expressed in MB, such as the insulin-like growth factor-I receptor, Tropomyosin-related kinase B (TrkB), Platelet-derived growth factor receptor-beta (PDGFRB), and c-Kit [33–35]. PI3K/Akt signaling has also been found to enhance the effects of Hedgehog in MB tumor cells and their cerebellar precursors [36, 37]. However, in the present study we did not detect co-expression of DJ-1 and p-Akt in the same tumor cell, which would have provided more direct evidence to elucidate their relationship and cooperative effect on the modulation of tumor progression. The precise mechanism of DJ-1, p-Akt, and related growth factor receptors in the development and progression of MBs will be investigated in further studies.

PTEN is another key negative regulator of the PI3K signaling pathway [38]. As MBs are known to display allelic losses of chromosomal region 10q, where the *PTEN* tumor suppressor gene is located, we hypothesize that inactivation of PTEN could constitute a possible mechanism that might be responsible for Akt activation. Recent data showed that losses of 10q are associated with metastatic disease at diagnosis [39], and the PTEN mutation detected in another study occurred only in a recurrent tumor that also carried a p53 mutation [40]. It seems that the frequency of *PTEN* mutations is low in MBs, and that mutation may be associated with a more aggressive or relapsing phenotype of the tumor. In the present study, we did not examine mutations of the *PTEN* gene. However, we found that PTEN was inactivated at the protein expression level. PTEN was significantly decreased in MB tumor cells compared with normal human cerebellum, indicating that inactivation of PTEN at the transcriptional level may also be involved in the regulation of PTEN and thus may influence the activation of Akt. Epigenetic inactivation of PTEN has recently been reported in several human tumors, including central nervous system tumors [41], but the related mechanisms of *PTEN* inactivation at the mRNA transcription level need to be clarified in further investigations.

In the current study, we found that high DJ-1 expression was closely associated with classic MBs, and the areas with high expression of DJ-1or p-Akt always coincided with the regions that had loss of PTEN expression. These regions were usually undifferentiated areas of classic MBs or internodular areas of desmoplastic MBs. However, expression of DJ-1 and p-Akt was decreased in intranodular areas of desmoplastic MBs, in which the tumor cells appeared to have varying degrees of neuronal differentiation. PTEN expression was also detectable in regions with neuronal differentiation. Therefore, we hypothesize that DJ-1and PTEN could modulate tumor cell differentiation via Akt pathway. DJ-1 is due to its effects on the PI3K signaling pathway, and may modulate PTEN function to produce hyperphosphorylation of Akt [6, 11]. Recent data have shown that PI3K/Akt signaling enhances the effects of Hedgehog in MBs and their cerebellar precursors, and that it may represent an indispensable precondition for the molecular realization of the Hedgehog signal [36, 37]. Inhibiting Hedgehog-dependent gene expression in MB can cause cell cycle arrest, consistent with the initiation of neuronal differentiation and loss of neuronal stem cell-like characteristics [42]. Molecular analysis has identified that the desmoplastic variant of MB is associated with activation of the Hedgehog-Patched signaling pathway [43]. The desmoplastic variant has been associated with a better prognosis than classic MB in some studies, although there is no significant difference in outcome for these two variants in our study. Thus, the DJ-1/PTEN/p-Akt/Hedgehog signaling system might play important roles in modulating cell differentiation and further influence the outcome of MBs. Thus, blocking cell growth and inducing cell differentiation in MBs by interfering with the DJ-1 protein and Hedgehog pathway might represent a novel approach to treat this tumor. In fact, some inhibitors of the Hedgehog pathway, such as cyclopamine and HhAntag, have been proven to cause the regression of murine tumor allografts *in vivo*, and induce rapid death of cells from freshly resected human MBs [44, 45].

## Conclusion

Although the precise roles of DJ-1, PTEN and p-Akt in MBs have not been identified, our study indicates links between these molecules in their protein expression level in MBs, and the validity of preliminary data in this study needs to be confirmed by a larger number of cases. We consider that DJ-1, PTEN, and p-Akt might play important roles in cell proliferation and differentiation of MBs, and their expression levels might be useful parameters for predicting the prognosis of patients with MB. Of course, more rigorous investigations are necessary to clarify their intrinsic links.
